# Predictive modeling of glioblastoma recurrence for therapeutic target identification

**DOI:** 10.1038/s44321-025-00236-0

**Published:** 2025-04-28

**Authors:** Hrvoje Miletic, Thomas Daubon

**Affiliations:** 1 Department of Pathology, Haukeland University Hospital, Bergen, Norway; 2 Department of Biomedicine, University of Bergen, Bergen, Norway; 3 University of Bordeaux, CNRS, IBGC, UMR5095 Bordeaux, France

**Keywords:** Cancer, Chromatin, Transcription & Genomics, Neuroscience

## Abstract

H Miletic and T Daubon discuss the study by S Lucchini et al, in this issue of *EMBO Mol. Med.*, which describes the generation and characterization of a novel model of glioblastoma recurrence to identify therapeutic vulnerabilities.

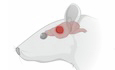

## Teaser

Patient-derived xenograft (PDX) models are an essential resource in cancer research, enabling the discovery of novel molecular mechanisms and therapeutic targets, while providing a preclinical platform for the evaluation of experimental treatments. For glioblastoma (GB), the most malignant primary brain tumor, PDX models have long remained artificial, being derived from serum-cultured and long-term cultured cell lines such as U87 and U251 (Huszthy et al, 2012). A major step forward was made in 2006 by Lee et al, who discovered that serum-free cultures in neural stem cell medium recapitulated patient tumors at the genotypic and phenotypic level much better than serum-derived cultures (Lee et al, 2006). This discovery set a new standard for the culture of patient-derived GB cells and their corresponding PDX models which are still used today. An alternative to cell culture in neural stem cell media is the development of PDX models through serial in vivo passaging of patient-derived 3D spheroids in immunodeficient animals. This model preserves the original genotype including EGFR amplification, which typically disappears in cell culture-derived models (Talasila et al, 2013).

GB standard treatment consists of surgery, radiation, and chemotherapy with Temozolomide (Stupp et al, 2005). Despite this treatment regimen, patient prognosis remains poor with a median survival of 15–18 months. Research over the past decades has focused on primary tumors and corresponding PDX models, however, all GB do recur and little progress has been made in identifying the molecular changes that drive recurrence. Recent studies have shed new light on tumor evolution after treatment by analyzing patient biopsies from recurrent tumors and comparing them with primary tumors (Tanner et al, 2024). However, experimental models to elucidate this situation are scarce. In 2020, Vaubel et al undertook a major effort to characterize 96 PDX models at the genomic and phenotypic level (Vaubel et al, 2020). Although this study included recurrent tumors, the authors focused more on a generalized comparison between PDX models and patient samples and did not focus on elucidating tumor evolution in pairs of primary and recurrent tumors. Golebiewska et al reported in the same year establishment and characterization of paired primary and recurrent PDX models of GB and IDH-mutated astrocytomas, a valuable resource for translational research (Golebiewska et al, 2020). More recently, Knudsen et al established surgical resection in PDX models. By comparing recurrent to primary tumors, they observed an increase in stemness properties at recurrence, which was mechanistically linked to upregulation of pleiotrophin expression in both tumor cells and macrophages/microglia (Knudsen et al, 2022).

**Figure 1:**
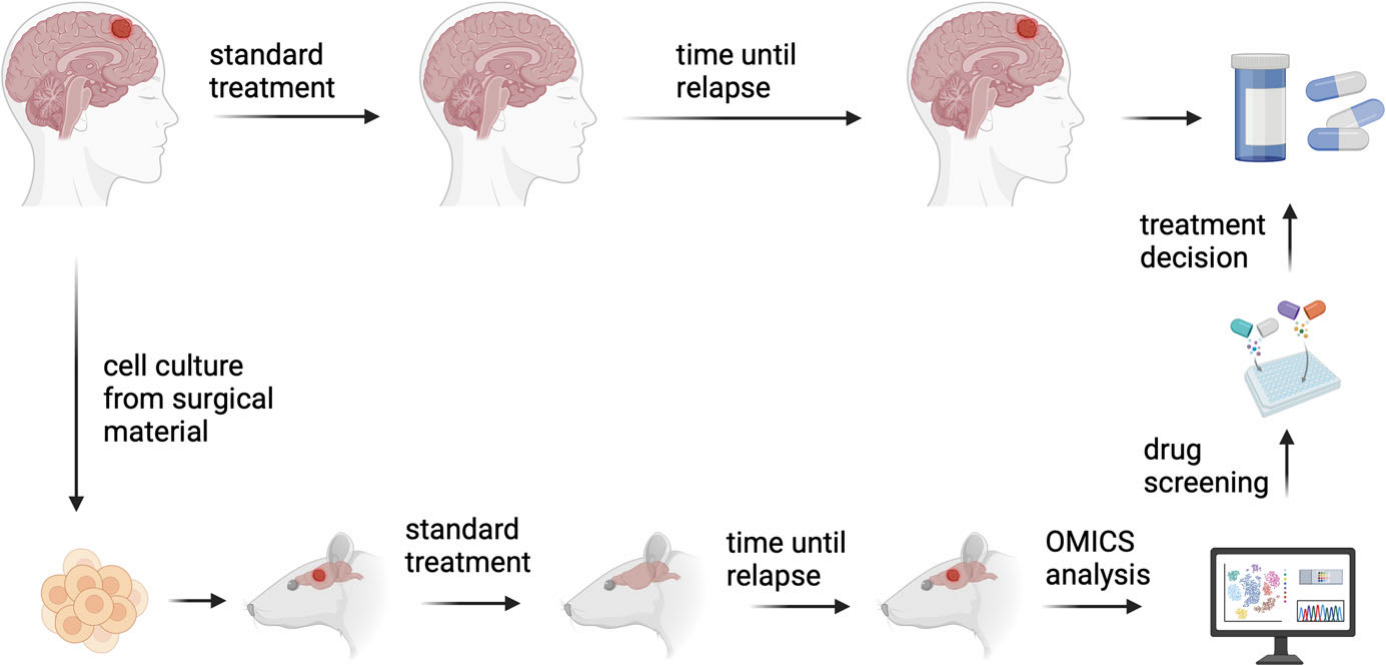
Schematic outline of induced-recurrence PDX model for GB which parallels development of recurrent tumors in patients. GB patients undergo surgical resection that provides biopsy material, which is used to create short-term cell cultures. These cultured cells are then implanted orthotopically into the brain of rodents to generate PDX models. The rodents receive treatments that mirror the standard clinical protocol for GB patients, including surgical resection, temozolomide chemotherapy, and radiation therapy. Following this standard treatment regimen, the rodents develop recurrent tumors, establishing what is referred to as an induced-recurrence PDX model in the study of Lucchini et al Combined OMICS analysis and drug screening can identify specific treatment targets/drugs that can be given to patients in the recurrence situation (created using Biorender).

In this issue of *EMBO Mol. Med.,* Lucchini et al established a new PDX model system for GB recurrence (Lucchini et al, 2025). Due to the fact that recurrent GB are not consistently removed by surgery, the researchers developed an induced recurrence model using PDX (IR-PDX). This model aims to predict the changes that occur during tumor recurrence in patients, potentially serving as a novel approach for precision medicine applications (Fig. 1). First, they established a recurrence PDX model based on primary and recurrent biopsies from 2 patients (GB39 and GB67), both with unmethylated MGMT promoter, and performed survival analyses as well as histological analyses confirming aggressive tumor growth with features seen in patient GB. To establish the IR-PDX model, the authors fine-tuned radiation dose and temozolomide dose in a MGMT methylated PDX model that is known to respond to this treatment regimen (GB80). They then performed needle injury, radiation, and chemotherapy on both patient-derived PDX models GB39 and GB67. Both models showed tumor volume reduction after treatment, followed by tumor recurrence. However, when comparing tumor volumes over time in the IR-PDX models compared to the primary tumors, there was no statistically significant difference in both the GB39 and GB67 models with unmethylated MGMT promoter. However, in the GB model with methylated MGMT promoter, there was a significant reduction in tumor volume with IR-PDX compared to the primary tumor. These data confirmed the clinical relevance of the IR-PDX model. This was further supported by NGS data showing an overlap of 37 mutations in both the recurrent and the IR-PDX models that were not present in the primary tumor. To verify the feasibility of a future precision medicine approach, the authors targeted a gain-of-function mutation in PIK3CA, present only in the recurrent models, with the drug Alpelisib. Viability was significantly reduced in cells derived from the recurrence model compared to primary tumor cells. Further molecular analyses revealed that >60% of gene expression changes and >30% of differentially methylated genes between primary and recurrent PDX were replicated in the IR-PDX model. GB often displays a switch towards a mesenchymal transcriptional subtype upon recurrence, which the authors verified with their IR-PDX model showing upregulation and hypomethylation of ZEB1, a transcription factor involved in epithelial-to-mesenchymal transition. The authors performed single-cell analyses on paired primary and recurrent tumors and on IR-PDX models, excluding mouse genes from the analysis. The results confirmed mesenchymal transition in recurrent samples, while IR-PDX models showed an intermediate transcriptional signature between primary and recurrent tumors. Notably, recurrent models displayed a distinct signature associated with cilia assembly, which was further validated in a large database (Wang et al, 2022). Cilia, which express specific components such as ARL13B and acetylated α-Tubulin, are typically associated with quiescent cells (Joiner et al, 2015). However, they also play a critical role in cell cycle regulation and act as signaling hubs, with receptor tyrosine kinases localizing to these structures. To investigate the functional role of cilia in recurrent tumor cells, models were treated with Mebendazole (MBZ) to pharmacologically inhibit ciliogenesis, resulting in reduced proliferation. Furthermore, the combination of MBZ and TMZ showed a synergistic effect, supporting the role of TMZ in promoting cilia formation in glioblastoma cells. Finally, the authors tested patient-derived cells with targeted drugs identified by scRNA-seq data analysis. Pyrimethamine and TRAM34, which inhibit SLC47A2 (Multidrug and Toxin Extrusion Protein 2) and KCNN4 (Potassium Calcium-Activated Channel Subfamily N Member 4), respectively, along with Zonisamide, an SCN4B sodium channel inhibitor, significantly reduced cell proliferation.

In this study, the authors developed a clinically relevant glioblastoma model using a straightforward approach that closely mimics clinical procedures. The needle injury used by the authors, which they describe as “mock surgery”, could be replaced in the future by a surgical procedure that more closely resembles a clinical scenario in which maximal safe resection is performed. The findings of Lucchini et al highlight the utility of the IR-PDX models for evaluating targeted therapies based on responses in recurrent tumors. Widespread adoption of such models in the neuro-oncology community could enhance the identification of therapeutic targets and facilitate the development of more effective treatments.

## References

[CR1] Golebiewska A, Hau AC, Oudin A, Stieber D, Yabo YA, Baus V, Barthelemy V, Klein E, Bougnaud S, Keunen O et al (2020) Patient-derived organoids and orthotopic xenografts of primary and recurrent gliomas represent relevant patient avatars for precision oncology. Acta Neuropathol 140:919–949 33009951 10.1007/s00401-020-02226-7 PMC7666297

[CR2] Huszthy PC, Daphu I, Niclou SP, Stieber D, Nigro JM, Sakariassen PO, Miletic H, Thorsen F, Bjerkvig R (2012) In vivo models of primary brain tumors: pitfalls and perspectives. Neuro Oncol 14:979–993 22679124 10.1093/neuonc/nos135 PMC3408261

[CR3] Joiner AM, Green WW, McIntyre JC, Allen BL, Schwob JE, Martens JR (2015) Primary cilia on horizontal basal cells regulate regeneration of the olfactory epithelium. J Neurosci 35:13761–13772 26446227 10.1523/JNEUROSCI.1708-15.2015 PMC4595624

[CR4] Knudsen AM, Halle B, Cedile O, Burton M, Baun C, Thisgaard H, Anand A, Hubert C, Thomassen M, Michaelsen SR et al (2022) Surgical resection of glioblastomas induces pleiotrophin-mediated self-renewal of glioblastoma stem cells in recurrent tumors. Neuro Oncol 24:1074–1087 34964899 10.1093/neuonc/noab302 PMC9248408

[CR5] Lee J, Kotliarova S, Kotliarov Y, Li A, Su Q, Donin NM, Pastorino S, Purow BW, Christopher N, Zhang W et al (2006) Tumor stem cells derived from glioblastomas cultured in bFGF and EGF more closely mirror the phenotype and genotype of primary tumors than do serum-cultured cell lines. Cancer Cell 9:391–403 16697959 10.1016/j.ccr.2006.03.030

[CR6] Lucchini S, Nicholson JG, Zhang X, Hoseham J, Lim YM, Mossner M, Millner TO, Brandner S, Graham T, Marino S (2025) A novel model of glioblastoma recurrence to identify therapeutic vulnerabilities. EMBO Mol Med 10.1038/s44321-025-00237-z 10.1038/s44321-025-00237-z PMC12162887 40295888

[CR7] Stupp R, Mason WP, van den Bent MJ, Weller M, Fisher B, Taphoorn MJ, Belanger K, Brandes AA, Marosi C, Bogdahn U et al (2005) Radiotherapy plus concomitant and adjuvant temozolomide for glioblastoma. N Engl J Med 352:987–996 15758009 10.1056/NEJMoa043330

[CR8] Talasila KM, Soentgerath A, Euskirchen P, Rosland GV, Wang J, Huszthy PC, Prestegarden L, Skaftnesmo KO, Sakariassen PO, Eskilsson E et al (2013) EGFR wild-type amplification and activation promote invasion and development of glioblastoma independent of angiogenesis. Acta Neuropathol 125:683–698 23429996 10.1007/s00401-013-1101-1 PMC3631314

[CR9] Tanner G, Barrow R, Ajaib S, Al-Jabri M, Ahmed N, Pollock S, Finetti M, Rippaus N, Bruns AF, Syed K et al (2024) IDHwt glioblastomas can be stratified by their transcriptional response to standard treatment, with implications for targeted therapy. Genome Biol 25: 45 38326875 10.1186/s13059-024-03172-3 PMC10848526

[CR10] Vaubel, Tian RA, Remonde S, Schroeder MA D, Mladek AC, Kitange GJ, Caron A, Kollmeyer TM, Grove R, Peng S et al (2020) Genomic and phenotypic characterization of a broad panel of patient-derived xenografts reflects the diversity of glioblastoma. Clin Cancer Res 26:1094–1104 31852831 10.1158/1078-0432.CCR-19-0909 PMC7056576

[CR11] Wang L, Jung J, Babikir H, Shamardani K, Jain S, Feng X, Gupta N, Rosi S, Chang S, Raleigh D et al (2022) A single-cell atlas of glioblastoma evolution under therapy reveals cell-intrinsic and cell-extrinsic therapeutic targets. Nat Cancer 3:1534–1552 36539501 10.1038/s43018-022-00475-x PMC9767870

